# Primary care physicians’ practice regarding diabetes mellitus diagnosis, evaluation and management in the West region of Cameroon

**DOI:** 10.1186/s12902-015-0016-3

**Published:** 2015-04-04

**Authors:** Ahmadou M Jingi, Jobert Richie N Nansseu, Jean Jacques N Noubiap

**Affiliations:** Department of Internal Medicine and Specialties, Faculty of Medicine and Biomedical Sciences, University of Yaoundé I, Yaoundé, Cameroon; Department of Public Health, Faculty of Medicine and Biomedical Sciences, Yaoundé, Cameroon; Internal Medicine Unit, Edéa Regional Hospital, Edéa, Cameroon; Medical Diagnostic Center, Yaoundé, Cameroon

**Keywords:** Primary care physicians, Diabetes mellitus, Sub-Saharan Africa, Cameroon

## Abstract

**Background:**

Primary care physicians (PCPs) are the main providers of diabetes care especially in resource-limited countries which experience extreme shortage of specialists. The present study aimed to evaluate PCPs’ approach towards diabetes mellitus (DM) diagnosis, evaluation and management in Cameroon.

**Methods:**

We carried-out a cross-sectional survey in February 2012 in the West Region of Cameroon. Using a structured pretested questionnaire, we interviewed all PCPs working in the region who were present at their working place when the investigators visited, and volunteered to be enrolled in the study.

**Results:**

Sixty-six PCPs were interviewed. Their ages ranged from 24 to 56 years (mean 38.3, standard deviation 9.2 years). The levels of knowledge of PCPs regarding DM diagnosis were: 72.7%, 37.9%, 19.7% and 32.8% respectively obtained when using fasting plasma glucose, post-prandial glycemia, random glycemia and glycated hemoglobin as diagnostic tools. Only 6 PCPs (9.9%) prescribed the correct minimal work-up to evaluate diabetes patients at diagnosis. PCPs advised lifestyle modifications in 92.4% of cases, and thirty nine (53.1%) PCP’s used to prescribe both generic and specialty oral anti-diabetic drugs in case of uncomplicated type 2 DM management. The two main classes of anti-diabetic drugs prescribed were biguanides (77.3%) and sulfonamides (60.6%). Nearly all PCPs (97%) used to give frequent follow-up appointments to their patients. Ninety eight point five percent of participants were willing to receive any further continuous training on DM management.

**Conclusion:**

PCPs knowledge and practices towards diabetes mellitus diagnosis, evaluation and management were not optimal, stressing the need to improve their capacities regarding diabetes care. As such, more educational initiatives should be taken on, alongside regular upgrade and dissemination of clinical guidelines.

## Background

The incidence and prevalence of diabetes mellitus (DM) especially type 2 diabetes mellitus (T2DM) has reached epidemic proportions worldwide, fuelled by population growth, aging, urbanization, and increasing prevalence of obesity and physical inactivity. Estimates from International Diabetes Federation (IDF) indicate that the number of adults with diabetes in the world will expand by 55%, from 381.8 million in 2013 to 591.9 million in 2035, with the largest increase in sub-Saharan Africa (SSA) [1]. DM causes significant morbidity, disability and early mortality through micro- and macro-vascular complications such as cerebrovascular disease, retinopathy, coronary heart disease, peripheral artery disease, nephropathy, and neuropathy [1]. Indeed in 2013, mortality attributable to diabetes in SSA accounted for almost 8.6% of total deaths recorded in the region [2]. Large studies such as the Diabetes Control and Complications Trial (DCCT), the U.K. Prospective Diabetes Study (UKPDS), and numerous other studies on cardiovascular risk reduction have demonstrated however that improved glycemic, blood pressure, and cholesterol control can substantially reduce the risk of complications emerging from DM [3-5].

In addition to growing numbers of individuals in whom DM has been diagnosed, in numerous others DM or prediabetes remains undiagnosed or is likely to develop in the near future. Identification of such individuals at risk for DM, as well as those who may already have the disease but in whom it has not yet been diagnosed is a key element in reducing the overall burden of the disease [6]. Early diagnosis and initiation of treatment can indeed prevent or delay disease progression and reduce consequently the risk for diabetes-related complications. Achieving evidence-based clinical goals by implementing effective management strategies substantially reduces the risk of morbidity and mortality, and ultimately improves patient outcomes [3-6].

As a matter of fact, primary care physicians (PCPs) are at the forefront of diabetes care as most of patients attend primary care services, especially in low-income countries where these services are ill-equipped to address the rising demand, experiencing also extreme shortage of specialists [7]. PCPs should therefore be highly capacitated to provide optimal diabetes care in order to prevent complications and improve quality of life of affected patients. In fact, lack of knowledge and inconsistent practice pertaining DM diagnosis, evaluation and management among care providers, long intervals between patient visits and limited consultation time due to overwhelming heavy patient load constitute major impediments to attainment of diabetes related goals of therapy [8,9]. The situation is further complicated by lack of access to a complete multidisciplinary diabetes health care team or by lack of systems within primary care practices to provide ongoing support for this chronic disease [10].

There is dearth of information on the aptitude of health care providers and particularly PCPs towards diabetes care in SSA. Therefore, this study aimed at assessing the knowledge and approach of PCPs to the diagnosis, evaluation and management of DM in the West region of Cameroon, a SSA country. It is anticipated that the study’s findings will contribute to build-up efficient strategies for optimal diabetes patients’ follow-up in primary health care facilities in the milieu.

## Methods

### Study design and participants

This was a cross-sectional study conducted in February 2012 in the West Region of Cameroon. This is one of the 10 administrative regions of the country, extending over 13,892 km^2^ of territory and hosting, in 2010, a population of 1,785,285 inhabitants [11]. It is divided into 20 health districts inside which are registered 530 health facilities both of the public and private sectors. The present study received approval from regional authorities of the Ministry of Public Health for the West Region, acting as the local Ethics Committee. All participants interviewed in the study provided a written informed consent before their inclusion.

We included all PCPs working in the region at the time of the survey irrespective of their age, gender, specialty, seniority, sector or location of practice, who were present at their work place when the investigators visited, and who consented to be enrolled in the study. There were 111 PCPs currently working in the region at the time of the study, of whom 66 responded to our inclusion criteria, the rest being absent from their work place when the investigators visited. Data were collected during an interview using a structured pretested questionnaire divided into 5 main items: background characteristics, knowledge on diagnosis of DM, evaluation of a diabetic patient at diagnosis, treatment and follow-up of diabetics. All the interviews were conducted by the same researcher who was a medical doctor. They were not audio-recorded, and PCPs had the opportunity to read the questions along with the interviewer.

### Background variables

Age was dichotomized into two groups: < 35 and ≥ 35 years. Location of practice was reported as “rural” or “urban”. Sector of practice was defined as “public” or “private”. Specialty of the physician was dichotomized, so that being a General Practitioner was coded “yes” and not being a General Practitioner, as “no”. Duration of practice was divided into two groups: < 10 and ≥ 10 years. The average number of patients seen per day was dichotomized in < 10 and ≥ 10 patients/day. Having previously taken part in any training on DM after graduation was reported as “yes” or “no” according to the answer given by the PCP.

### Knowledge on the diagnosis of diabetes

Eleven multiple choice questions constituted this item, investigating the different cut-off values to define or diagnose diabetes and hypertension in patients living with diabetes mellitus. Levels of Fasting Plasma Glucose (FPG) to diagnose diabetes and fasting hyperglycemia were dichotomized as “correct” when PCPs answered 1.26 g/l and 1.1 g/l respectively, and “incorrect” when given another answer. Levels of Post-prandial Glycemia (PPG) to define diabetes and impaired glucose tolerance were reported as “correct” if PCPs answered 2.0 g/l and 1.4 g/l respectively, and “incorrect” when another answer was given. Levels of random glycemia to define diabetes were dichotomized as that of PPG. Levels of Glycated Hemoglobin to define diabetes were defined as “correct” when the PCP answered 6.5% and “incorrect” in case he/she gave a different answer. Levels of systolic/diastolic blood pressure to define hypertension in diabetes patients or those with chronic kidney disease were dichotomized in “correct” when PCPs answered 130/80 mmHg, and “incorrect” if given a different answer. These definitions were in keeping with WHO, American Diabetes Association (ADA) and Joint National Committee (JNC 7) guidelines [12-14].

### Initial evaluation of diabetic patients

We asked participants to list morphologic and biological exams they used to ask when a patient is diagnosed with diabetes. Each exam was considered adequate or not based on initial evaluation of diabetes according to ADA recommendations [13].

### Treatment and follow-up of diabetic patients

Questions targeted patient education and advices towards non-pharmacological measures; drug prescriptions (either mono- or combined-therapy); completeness of physical examination of a patient with diabetes; rhythm of follow-up appointments; source of information to up-date knowledge on diabetes care, and willingness to take part in future continuous trainings on DM. We reported completeness of diabetes patients’ physical examination as “complete” when they systematically measured blood pressure, weight, waist circumference, and examined the feet, and “incomplete” if at least one of these measures was lacking.

### Statistical methods

Data analysis used Statistical Package for Social Sciences (SPSS) version 20.0 (IBM Corp. Released 2011. IBM SPSS Statistics for Windows, Version 20.0. Armonk, NY: IBM Corp.). Results are expressed as count (proportion) or mean (Standard Deviation) as appropriate. The Chi-2 test was used for categorical variable comparisons. Odds Ratios (OR) with 95% Confidence Intervals (CI) were used to assess the impact of background variables on different outcomes, and were calculated by both univariate and multivariate logistic regressions while adjusting for confounders. Multivariate logistic regressions used the stepwise forward method, and only variables with a p value < 0.25 in univariate analyses were introduced in the model. A *p* value < 0.05 was used to characterize statistically significant results.

## Results

### Background characteristics

Sixty-six out of 111 PCPs were interviewed (i.e. a response rate of 59.5%), mostly men (69.7%). Ages ranged from 24 to 56 years with a mean of 38.3 (9.2) years. Table 1 displays the different background characteristics of our respondents. Most of PCPs were located in an urban area (69.7%), and 37 (56.1%) of them were practicing in public health facilities. Fifty six (84.6%) PCPs were General Practitioners. Duration of practice varied from 0 to 26 years, with a mean of 9.5 (7.8) years. The number of patients seen per day ranged from 1 to 40, with a mean of 10.8 (6.2) patients/day. Specifically, participants used to see 0 to 10 diabetes patients per day, with an average of 1.7 (1.4) patients/day. Sixty two percent of them have previously attended special courses or trainings targeting diabetes care after their graduation.

**Table 1:** **Background characteristics of the study population**

**Characteristic**	**Number (N = 66)**	**Percentage (%)**
Age groups		
<35 years	20	30.3
≥35 years	46	69.7
Sex		
Male	46	69.7
Female	20	30.3
Sector of practice		
Public	37	56.1
Private	29	43.9
Area of practice		
Urban	46	69.7
Rural	20	30.3
Specialty of the physician		
General Practitioner	56	84.8
Surgeon	1	1.5
Gynecologist-obstetrician	1	1.5
Internist	1	1.5
Pneumologist	1	1.5
Pediatrician	1	1.5
Public Health specialist	5	7.7
Duration of practice		
<10 years	33	50
≥10 years	33	50
Average number of patients seen per day		
<10 per day	24	36.4
≥10 per day	42	63.6
Training on DM after graduation		
Yes	41	62.1
No	25	37.9

DM = Diabetes Mellitus.

### Definition of diabetes mellitus

Forty eight (72.7%) of our respondents knew what was the exact level of FPG to define DM, but only 6 (9.1%) of them gave the right level of FPG to define fasting hyperglycemia. Only 25 (37.9%) PCPs gave the right threshold of PPG to define DM, and 18 (27.3%) participants knew what was the exact level of PPG to define impaired glucose tolerance. Likewise, levels of random glycemia to define diabetes were correct in only 19.7% of cases. Fifty eight (87.9%) PCPs knew that Glycated Hemoglobin can be used for the diagnosis of diabetes among whom only 19 (32.8%) gave the correct related level to define DM. Only 23 (34.8%) and 17 (25.8%) participants correctly defined diagnosis thresholds of hypertension in diabetes with regard to systolic and diastolic blood pressures respectively (see Table 2). We found no relationship between knowledge of PCPs on levels of FPG to define DM and background variables (see Table 3).

**Table 2:** **Knowledge on diabetes, its diagnosis, evaluation and management**

	**Number (N = 66)**	**Percentage (%)**
Level of FPG to define DM: correct	48	72.7
Level of FPG to define fasting hyperglycemia: correct	6	9.1
Level of PPG to define DM: correct	25	37.9
Level of PPG to define impaired glucose tolerance: correct	18	27.3
Level of random glycemia to define DM: correct	13	19.7
Can Glycated Hemoglobin be used to define diabetes: Yes	58	87.9
Level of Glycated Hemoglobin to define diabetes (N = 58): correct	19	32.8
Level of systolic blood pressure to define hypertension in a patient with DM: correct	23	34.8
Level of diastolic blood pressure to define hypertension in a patient with DM: correct	17	25.8
Frequently asked morphologic and biological exams		
Full blood count	31	47.0
Serum urea	49	74.2
Creatinemia	55	83.3
Serum uric acid	24	36.4
Urinary dip stick	27	40.9
Total cholesterol	33	50.0
HDL cholesterol	43	65.2
Triglycerides	28	42.4
Natremia	12	18.2
Kaliemia	19	28.8
Chloremia	12	18.2
Calcemia	13	19.7
Magnesemia	9	13.6
Chest X ray	7	10.6
Cardiac echography	5	7.6
Echocardiogram	18	27.3
Fundoscopy	18	27.3
Correct (minimal) workup asked	6	9.9
Used to recommend lifestyle modifications: Yes	61	92.4
Used to refer diabetes patients to the nutritionist: Yes	46	69.7
Physical examination during diabetes patients’ consultations: complete	24	36.4
Type of drug usually prescribed in type 2 diabetes mellitus		
Generic	14	21.2
Specialty	9	13.6
Both	35	53.1
Missing data	8	12.1
Class of drugs frequently prescribed as first line treatment in type 2 diabetes mellitus management		
Biguanides	51	77.3
Sulfonamides	40	60.6
Glinids	5	7.6
Glitazones	3	4.5
Insulin	1	1.5
Alpha-glycosidase inhibitors	2	3.0
DPP-4 inhibitors	1	1.5
Used to prescribe 2 or more anti-diabetic drugs in patients with uncomplicated type 2 diabetes mellitus: Yes	34	51.5
Combinations usually prescribed (N = 34)		
Sulfonamide-Biguanide	31	91.2
Sulfonamide-Glinid	1	2.9
Sulfonamide-Insulin	3	8.8
Biguanide-Glinid	3	8.8
Biguanide-Insulin	1	2.9
Biguanide-Alpha-glycosidase inhibitors	1	2.9
Biguanide-DPP-4 inhibitors	1	2.9
Type of drugs usually prescribed in patients presenting with a hyperglycemic emergency		
Oral anti-diabetics	7	10.6
Insulin	57	86.4
Missing data	2	3.0
Used to manage comorbidities: Yes	6	9.1
Follow-up appointments usually given to diabetes patients: Yes	64	97.0
Frequency of appointments (N = 62)		
Every week	15	22.7
Every two weeks	17	25.8
Once in a month	32	48.5
Usually seek for information on new guidelines regarding diabetes care: Yes	51	77.3
Source of information (N = 51)		
Medical visitor	39	76.5
Conferences	20	39.2
Colleague	20	39.2
Internet	14	27.5
Scientific journals	9	17.6
Willingness to participate in future workshops on DM: Yes	65	98.5

DM = Diabetes Mellitus; FPG = Fasting Plasma Glucose; PPG = Post-prandial Glycemia.

**Table 3:** **Factors influencing incorrect definition of diabetes mellitus with regard to the level of fasting plasma glucose, no recommendation of lifestyle modifications, and incomplete physical examination during diabetes patients’ consultation in univariate analyses**

**Characteristic**	**Incorrect definition of DM**		**Lifestyle modifications**		**Physical examination**	
	**OR (95% CI)**		**OR (95% CI)**		**OR (95% CI)**	
		***p***		***p***		***p***
Age						
<35 years	1		1		1	
≥35 years	2.02 (0.62-6.57)	0.237	1.083 (0.37-3.14)	0.883	2.64 (0.94-7.43)	0.063
Sex						
Male	1		1		1	
Female	0.57 (0.16-2.02)	0.382	1.23 (0.40-3.75)	0.714	0.59 (0.20-1.73)	0.336
Sector of practice						
Public	1		1		1	
Private	0.75 (0.25-2.27)	0.613	0.523 (0.18-1.54)	0.236	0.52 (0.19-1.44)	0.206
Area of practice						
Urban	1		1		1	
Rural	0.85 (0.26-2.81)	0.785	1.23 (0.40-3.75)	0.714	1.09 (0.36-3.26)	0.879
General practitioner						
Yes	1		1		1	
No	2 (0.49-8.13)	0.327	1.53 (0.38-6.13)	0.396	2.59 (0.50-13.34)	0.212
Duration of practice						
<10 years	1		1		1	
≥10 years	1.36 (0.46-4.04)	0.580	0.87 (0.31-2.45)	0.792	3.95 (1.34-11.60)	0.011*
Average number of patients seen per day						
<10	1		1		1	
≥10	0.94 (0.31-2.87)	0.917	0.41 (0.14-1.19)	0.097	0.74 (0.26-2.10)	0.565
Training on DM after graduation						
Yes	1		1		1	
No	0.36 (0.10-1.11)	0.070	1.33 (0.45-3.95)	0.603	0.55 (0.19-1.60)	0.270

*p value < 0.05.

### Initial evaluation of diabetes

Fifty seven (86.4%) and 60 (90.1%) PCPs were used to asking some morphologic and biological exams when they have diagnosed a patient with type 1 and type 2 DM respectively. The most frequently asked exams were: creatinemia (83.3%), serum urea (74.2%), HDL cholesterol (65.2%), Total cholesterol (50%), full blood count (47%), triglycerides (42%), and urinary dip stick (40.9%). Only 6 PCPs (9.9%) did correctly prescribe the minimal work-up to evaluate a diabetes patient at diagnosis in keeping with ADA recommendations (see Table 2).

### Treatment

PCPs used to recommend lifestyle modifications in 92.4% of cases. This attitude was not influenced by any of the aforementioned background characteristics: all p values > 0.05 (see Table 3). Not more than 24 (36.4%) of our respondents performed a complete physical examination during diabetes patients’ consultations. PCPs who have been practicing for ten years and above had 3.95 more “chances” to incompletely examine diabetes patients (95% CI: 1.34 – 11.60; p = 0.011), but this odds was no more significant after adjustment [(adjusted odds ratio 4.7, 95% CI: 0.83 – 26.73; p = 0.081) (see Table 4)]. The other background variables did not influence the completeness of diabetes patients’ physical examination (see Table 3). Thirty nine (53.1%) PCP’s were used to prescribing both generic and specialty oral anti-diabetic drugs in case of uncomplicated T2DM management. The two main classes of drugs frequently prescribed were biguanides (77.3%) and sulfonamides (60.6%), either in mono- or in combined-therapy (see Table 2). Besides, 57 (86.4%) PCPs used insulin to manage hyperglycemic emergencies, while 7 (10.6%) used oral anti-diabetic drugs in similar situations (see Table 2).

**Table 4:** **Factors influencing incorrect definition of diabetes mellitus with regard to the level of fasting plasma glucose, no recommendation of lifestyle modifications, and incomplete physical examination during diabetes patients’ consultation after adjustment**

**Characteristic** ^*^	**Incorrect definition of DM**		**Lifestyle modifications**		**Physical examination**	
	**OR (95% CI)**		**OR (95% CI)**		**OR (95% CI)**	
		***p***		***p***		***p***
Age						
<35 years	1		/		1	
≥35 years	1.54 (0.44-5.37)	0.495			1.54 (0.27-8.70)	0.627
Sector of practice						
Public	/		1		1	
Private			0.25 (0.02-2.62)	0.248	0.51 (0.17-1.53)	0.230
General practitioner						
Yes	/		/		1	
No					1.82 (0.28-11.77)	0.529
Duration of practice						
<10 years	/		/		1	
≥10 years					4.7 (0.83-26.73)	0.081
Average number of patients seen per day						
<10	/		1		/	
≥10			0.61 (0.08-4.35)	0.618		
Training on DM after graduation						
Yes	1		/		/	
No	0.41 (0.13-1.32)	0.135				

*We introduced in the model all variables that had a p value < 0.25 in univariate analyses.

### Follow-up

Only 6 (9.9%) participants used to care about managing comorbidities concurrently with diabetes management. Sixty four (97.0%) PCPs used to give regular follow-up appointments to their diabetes patients, the most encountered frequency being once in a month (48.5%) (see Table 2). Fifty one (77.3%) PCPs were used to constantly seeking information on new guidelines regarding diabetes care, the 3 dominating sources of information being medical visitors (76.5%), conferences (39.2%), and colleagues (39.2%) (see Table 2). Ninety eight point five percent of our respondents were willing to take part in future continuous medical education targeting DM.

## Discussion

This study shows that the majority of PCPs knew the DM diagnostic criteria for FPG (72.7%); however, fewer PCPs knew the criteria for other glycemic parameters such as PPG, random glycemia and HbA1c (37.9%, 19.7%, and 32.8% respectively). Very few PCPs (9.9%) prescribe the correct minimal workup at diagnosis thereby seeking for comorbidities. Almost all PCPs (92.4%) do advise lifestyle changes to their diabetes patients and give regular follow-up appointments as well (97% of cases). The two main classes of drugs frequently prescribed are biguanides (77.3%) and sulfonamides (60.6%). Duration of practice may be an independent factor impacting our PCPs completeness of their diabetes patients’ physical examination. Consequently, regular continuous training sessions must be organized for PCPs on DM diagnosis and management, and the right information must be made available to them so as to enhance their ability to provide optimal DM care.

Although we found that more than half of our respondents (62%) have already participated in special trainings on DM, this factor did not influence PCPs knowledge on DM diagnosis, evaluation and management. It is therefore questionable how these courses are being delivered and at what rhythm they are organized. One should however bear in mind that our PCPs were adults. We found for instance a mean age of 38 years. Therefore, the teaching methods should be adapted accordingly as it has recently been proposed by Tamasik et al. [15]. Happily, 65 (98.5%) PCPs expressed the willingness to take part in more trainings focused on DM to increase and strengthen their knowledge and skills.

Identification of individuals at risk for DM especially T2DM, as well as of those who may already have the disease but in whom it has not yet been diagnosed, is a key element in reducing the overall burden of the disease [6]. Further, early initiation of treatment can prevent or delay disease progression and reduce the risk for diabetes-related complications [3-6]. DM remains underdiagnosed [6] and appears as a complex chronic disease requiring lifelong self-management and continuous medical care to prevent its acute complications and reduce its associated chronic health risks [13]. PCPs have been recognized as responsible for provision of care to diabetes patients [15]. They must be consequently equipped with valuable tools to correctly diagnose and manage patients suffering from DM. If the majority of our PCPs knew how to diagnose DM using FPG (72.7%), very few knew, contrariwise, how to identify pre-diabetic states and diagnose diabetes using other glycemic parameters, namely PPG, random glycemia and Glycated Hemoglobin (37.9%, 19.7%, and 32.8% respectively). Accordingly, it has been clearly pointed out that many patients with impaired fasting glucose levels, impaired glucose tolerance, or both conditions are already experiencing the consequences of micro-vascular disease, including blindness, amputations (due to neuropathy and infection), and kidney failure [16]. PCPs must thereby be well trained at seeking and identifying prediabetes for an early and efficient intervention.

Several studies have demonstrated that glycemic control in diabetes patients must be accompanied by efficient management of comorbidities. In fact, early assessment and control of diabetes, alongside adequate control of blood pressure and lipid levels, may delay progression of vascular complications and improve consequently patients’ outcome [3-6,13]. It appears thus of great importance to seek for, and manage other metabolic and cardiovascular conditions that can affect patient prognosis. Although 57 (86.4%) and 60 (90.9%) PCPs were used to asking morphologic and biological exams to diabetes patients in cases of type 1 and type 2 DM respectively, only 6 (9.9%) of them were used to asking for the minimal workup recommended by ADA [13], and cared as well for the identification and control of comorbidities. This could perhaps be justified by the fact that many primary care clinics of the West region of Cameroon are ill-equipped and do not permit to have all the required exams performed *in situ*, precluding thereby PCPs from an exhaustive work-up. Additionally, PCPs may be influenced by the financial resources of the patient, and so will prescribe few exams to patients with limited financial resources. These results suggest perhaps, on another hand, that our PCPs may not be familiar with international guidelines with respect to evaluation of diabetes patients and control of comorbidities. For instance, few PCPs (<35%) were aware of the levels of systolic or diastolic blood pressure to define hypertension in diabetic patients and those suffering from chronic kidney disease. It is true nonetheless that there are currently no national guidelines in the domain.

Management of DM integrates both lifestyle changes and anti-diabetic medication (either oral drugs or insulin). In this survey, 61 (92.4%) PCPs used to counsel lifestyle modifications to their patients, specifically meal planning plus regular physical exercise. This is similar to the 90.3% proportion lately reported by Morishita et al. in Japan [17]. Indeed, exercise counseling is very important for the prevention and inhibition of progression of many chronic diseases, including metabolic syndrome and cardiovascular diseases [18-20]. Increased physical activity has been reported to have beneficial effects on the incidence and prognosis of these chronic diseases, as well as overall morbidity and mortality [18-20]. Our PCPs seem to know how important must be the place occupied by lifestyle changes in DM management. It is interesting to mention that recent data have figured out a positive association between PCPs’ own exercise habits and recommendation of exercise in cases of hyperlipidemia, heart failure and hypertension, this becoming untrue in cases of apoplexia and DM [17]. Besides, other studies have also bolstered that physicians own lifestyle may influence the lifestyle counseling directed to their patients [21,22]. PCPs should therefore be encouraged to adopt a healthy lifestyle so that they can easily counsel their patients on the merits of such a habit.

The consensus statement of the American Diabetes Association (ADA) and the European Association for the Study of Diabetes (EASD) recommends simultaneous initiation of metformin and lifestyle intervention at diagnosis [23]. Metformin has the advantage of being relatively cheaper and presents very few adverse effects [13]. The two main oral anti-diabetic drugs our PCPs used to prescribe were biguanides (77.3%) and sulfonamides (60.6%), this result being consistent with that of Spann et al. (54.1% and 53.3% respectively) in primary settings in the United States [24]. Other classes of drugs such as alpha-glucosidase inhibitors and thiazolidinediones were scarcely used by our respondents. The most common prescribed association was biguanide-sulfonamide (91.2%), and we noticed that 10.6% of participants wrongly use oral anti-diabetic drugs instead of insulin to manage diabetes emergencies. PCPs should therefore be trained on how to manage diabetes emergencies.

Ninety seven percent of our PCPs usually gave follow-up appointments to their diabetes patients, the most encountered frequency being once in a month. Many patients need to return frequently for office visits in the beginning of their treatment. Once the management process is established, a good rule of thumb for return visits is every 3 months [6]. Moreover, the algorithms for the management of T2DM developed by the ADA/EASD place an increased emphasis on tight glycemic control and call for the assessment of patients every 2 to 3 months with a switch to new regimens or the addition of medications when glycemic goals are not met [23].

Although we did not ask to PCPs how much time they spent with diabetes patients during consultations, we can hypothesize that this time is not that so much, given their volume of work. We have seen for instance that some PCPs have to see not less than 40 patients per day. Besides, only 24 (36.4%) PCPs completely examined their diabetes patients during consultations, PCPs having been practicing for 10 years and above showing a 4 times increased likelihood to incompletely examine their patients. Does the seniority impede the quality of care to diabetes patients is therefore a question that needs further considerations. But to successfully manage diabetes, patients may need more contact with the care team than a single PCP can provide [7]. Some studies have proposed the incorporation of specialized nurses and pharmacists in order to be closer to diabetes patients so as to help them reaching their treatment goals [6,7,25]. Such associations need to be experimented in our settings.

Unfortunately, this study presents some limitations, these being inherent in practice-based research and cross-sectional descriptive works of this kind. First, due to our sampling method, we obtained a relatively low response rate (59.5%), hence a low sample size and possibly related statistically un-significant results. Second, our results cannot be generalized to the entire Cameroonian PCPs’ population, as we recruited only PCPs working in the West region of the country, and they may have differed from the larger macrocosm of practicing PCPs. Nonetheless, this is the first study undertaken in the milieu devoted at evaluating PCPs’ knowledge and practices regarding DM diagnosis and management. Further studies need to be conducted with larger samples to better elucidate the challenges faced by PCPs to achieve optimal diabetes care and their adherence to guidelines for the treatment and management of diabetes mellitus.

## Conclusion

In the West Region of Cameroon, PCPs knowledge and practices towards diabetes mellitus diagnosis, evaluation, treatment and follow-up are not optimal at all. This situation needs an urgent improvement and enhancement in order to maximize PCPs capabilities to deliver adequate and efficient quality of care to diabetes patients. As such, more educational initiatives like workshops or continuous trainings should regularly be taken on, and new teaching methods, appropriate for adults, should be implemented. Additionally, regular upgrade and dissemination of clinical guidelines should be undertaken by independent professional organizations.
